# Artificial Versus Magneto-Aerotactic Bacterial Robots for Cancer Therapy

**DOI:** 10.3390/mi17091026

**Published:** 2026-08-28

**Authors:** Sylvain Martel

**Affiliations:** NanoRobotics Laboratory, Department of Computer and Software Engineering, Institute of Biomedical Engineering, Polytechnique Montréal, Montréal, QC H3T 1J4, Canada; sylvain.martel@polymtl.ca

**Keywords:** microscale robots, cancer, hypoxia, magnetic field, magnetotactic bacteria

## Abstract

The use of magnetotactic bacteria (MTB), proposed by the author about 25 years ago, allowed the implementation of embedded capabilities allowing the execution of specific complex tasks such as the target delivery of therapeutics in tumoral hypoxic zones that could not be implemented in artificial micro/nanorobots due to technological limitations at such a small scale. Since then, technology has evolved but the question remains about how long such superior advantage of using only certain types of live microorganisms will last before embedding such capabilities in artificial micro/nanorobots becomes feasible while being a viable alternative. Although it is difficult to answer this question, this paper aims to provide clues with a perspective view about the challenges of implementing an artificial or synthetic counterpart by providing a general description of an equivalent artificial implementation of the embedded functional units found in such magneto-aerotactic bacterial robots for cancer therapy.

## 1. Introduction

Magnetotactic bacteria (MTB) [1] such as the MC-1 [2] are being used as a critical component of biohybrid microscale robots dedicated to cancer therapy. Each cell is self-propelled and has a chain of membrane-based nanoparticles known as magnetosomes. Such a chain behaves similarly to a magnetic compass needle and when a directional magnetic field is applied, a directional torque is induced on the chain of magnetosomes that guides the bacteria along the lines of magnetic field while being influenced by the oxygen gradients [3] along the displacement path to reach regions characterized with a specific range of target oxygen levels (TOL). Alone, these characteristics found in such magneto-aerotactic bacteria motivate the use of such microorganisms for the implementation of suitable microscale robots to target and deliver therapeutics in tumoral hypoxic zones [4], potentially improving substantially treatments for approximately 85% of all cancers.

Tumor hypoxic zones [5] are characterized by a lower level of oxygen. The formation of such hypoxic zones occurs when the rate of proliferating cancer cells outgrows the formation of neovascular vessels acting as the source of oxygen needed for the survival of the cancer cells. The combination of the resulting oxygen gradient and the consumption of available oxygen by the cancer cells create such hypoxic zones that are generally located from approximately 70 to 180 µm from the tumor vasculature.

This lower oxygen level makes radiotherapeutic treatments much less efficient [6] while the relatively high distance and the available diffusion gradient limit the capability of chemotherapeutic agents to reach hypoxic cells. Therefore, modern cancer therapies are still relatively inefficient at treating tumoral hypoxic regions [7,8,9]. Such a fact suggests that propelled microscopic agents, such as microrobots, could provide a solution to the diffusion limitation of chemotherapeutics. In a clinical setting, inducing sufficient propelling force using an external magnetic source would be limited by the overall volume magnetization of microscopic agents where the size must be sufficiently small for efficient intercellular displacement. This suggests that the use of self-propelled microscopic robots would be a more adequate solution to allow efficient travel in such a tumoral environment.

But besides the treatment of hypoxic cells, there is another advantage at targeting tumor hypoxic zones which is specificity. Indeed, due to limited spatial resolution and contrast enhancement, medical imaging modalities are often inadequate at providing reliable information to locate all cancer cells. Since hypoxic zones have oxygen levels that are much lower than the levels encountered in proximity of the tumor, taking advantage of this specificity by targeting hypoxic zones would allow efficient and reliable distribution of therapeutics throughout the whole tumor, provided that the self-propelled microscopic robots could deliver sufficient therapeutic payloads to yield a diffusion gradient to allow all cancer cells to receive the required dose. The fact that medical imaging modalities are also inefficient at providing adequate information to target hypoxic zones calls for more autonomous microscopic robots. More precisely, the implementation of such highly effective microscale robots must rely, among other components, on the proper integration of a system with the capability to target such hypoxic zones autonomously.

## 2. Hypoxia Targeting System (HTS)

Specificity of tumor hypoxic zones includes not only a lower oxygen level but also a more acidic environment, hence characterized by a lower pH. Such a change in the level of pH is due to the metabolic adaptation of hypoxic cells consuming glucose to compensate for the lack of oxygen. Although the pH level could be used by microscale robots to target tumor hypoxic zones, once the hypoxic cells are eliminated, the pH may not remain sufficiently low to provide the specificity required for subsequent autonomous targeting, if required. On the other hand, the elimination of such hypoxic cells does not significantly change the level of oxygen since hypoxic cells do not primarily consume oxygen, hence maintaining the specificity required for additional treatments.

The general method used by MTB to target regions of low oxygen in their natural environment can also be applied to target tumor hypoxic zones: first, these magneto-aerotactic bacteria sample using specific frequencies, and the O_2_ level along the lines of magnetic field and towards the north pole if north-seeking (NS) polar bacteria are used. The zones targeted by these MTB seeking specific TOL are referred to as the oxic-anoxic transition zones (OATZ), also being referred to as oxic-anoxic interface (OAI). Inside a tumor, the OATZ are located within the range of oxygen levels characterizing tumor hypoxic zones.

When outside a tumor hypoxic zone, the bacteria will move forward towards the next OATZ if above TOL and the second sample indicates a lower O_2_ level, hence corresponding to a decreasing oxygen gradient. While remaining above TOL, motion towards the last transited OATZ will occur if the second sample indicates a higher O_2_ level, hence corresponding to an increased oxygen gradient. When below TOL, the direction of motion to target the OATZ will be the reciprocal of the direction of motion used when above TOL. When successive samples regarding the sensitivity of the oxygen sensors of the bacteria indicate identical levels of O_2_, it will be interpreted as an absence of an oxygen gradient where forward or backward motion could be envisioned.

**Figure 1:**
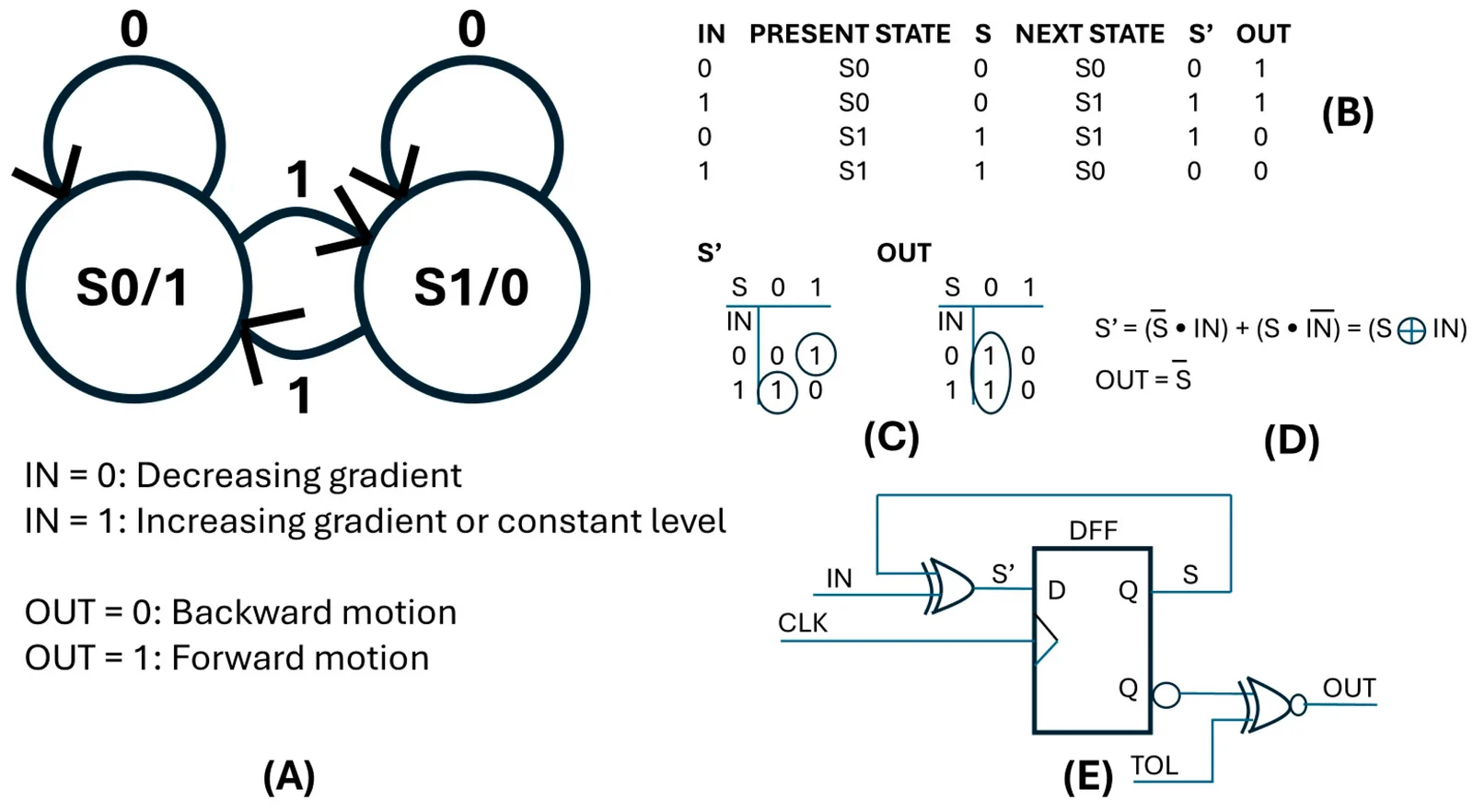
A simple example of a potential digital electronic circuit implementing an FSM describing a method like the one used by magneto-aerotactic bacteria to target regions of low oxygen such as tumor hypoxic zones. (**A**) State diagram of the HTS for operations at or above TOL (TOL = 1); (**B**) Corresponding state table; (**C**) Corresponding Karnaugh maps; (**D**) Corresponding Boolean expressions using the minterms for the output OUT and the next state S’ rewritten with the equivalent exclusive-OR (XOR) operation; (**E**) Resulting HTS circuit using a D-type flip-flop (DFF) as a state-memory and an XOR gate to determine the next state S’ from the present state S and the input IN. An exclusive-NOR (XNOR) gate has been added to act as an inverter to reverse the direction of displacement when TOL = 0, indicating operations below TOL.

To appreciate the complexity and feasibility of implementing such hypoxia-targeting systems in a hypothetical artificial microscale robot, an engineering methodology would first describe such behavior as a finite-state machine (FSM) as depicted in Figure 1A. It should be mentioned that the general implementation described here is used to provide an idea about the level of complexity involved and, therefore, does not represent the only design option.

The FSM here assumes a digital voltage input IN representing 0 and 1 for a decreasing and increasing gradient or constant oxygen level, respectively. This level is determined by another electronic system referred to here as an oxygen gradient detector (OGD) that mimics the input coming from the oxygen sensors of MTB. Similarly, another input, designated TOL, indicates if levels are below, at or above TOL when it is 0 or 1, respectively. The more aggressive tumors will have necrotic zones. Such necrotic zones are surrounded by hypoxic zones and since necrotic zones are located further away from the neovascular vessels compared to hypoxic zones, the level of oxygen in necrotic zones will be lower than TOL, hence the need for the HTS to check if levels are below, at or above TOL to set the direction of motion to target hypoxic zones. The output OUT of the circuit controlling the directional displacement that is set here to 0 or 1 is sent to an electronic controller or interface connected to a propelling system allowing a backward or forward displacement along the lines of magnetic field, respectively. Displacement along the lines of magnetic field can be achieved relatively easily with the implementation of a structure such as a microscopic magnetic compass needle acting like the chain of magnetosomes found in MTB.

The resulting FSM depicted in Figure 1A is relatively simple, with only two states designated here as S0 and S1. At S0, the output OUT is set to 1 for forward motion. A clock (CLK) set at a specific frequency synchronizes the whole process. The input is verified at each period of the clock, generally at the rising edge of CLK. At S0, when the input IN is 0 indicating a decreasing oxygen gradient, the system remains at S0 as shown by the arrow pointing toward S0, hence continuing with a forward motion if at or above TOL; otherwise, the direction of motion is reversed. While remaining above TOL, when the input IN changes to 1, indicating an increasing gradient or constant oxygen level, the system switches to S1, while setting OUT to 0 to initiate a backward displacement. Backward displacement along the lines of magnetic field will continue at S1 as long as IN = 1 when still above TOL. When IN switches to 0, indicating a decreasing oxygen gradient, the FSM switches back to S0, reversing to a forward motion if above TOL; otherwise, if below TOL, it will continue with a backward motion.

Figure 1B depicts the equivalent state table with S and S’ representing the present state and the next state, respectively. The simplified Boolean expression for S’ and OUT can be deducted from simple Karnaugh maps, as shown in Figure 1C, where a sum of products (minterms) has been selected as an example here instead of the equivalent product of sums (maxterms). A bar over the signal name indicates an inversion, a dot represents an AND gate, a plus sign represents an OR gate, and a circle around a plus sign indicating an exclusive-OR gate.

The resulting logic circuit is depicted in Figure 1E where a D-type flip-flop (DFF) acts as the state memory. Other alternative circuits are also possible. As mentioned earlier, this example is used only to provide an idea about the level of complexity involved in implementing such a hypoxia-targeting system that could be considered the ‘brain’ of such a microscale robot. It may be surprising to see how simple this system is. But the level of complexity increases when other required components are considered, starting with the OGD mentioned earlier.

Unlike in the natural environment, the decrease of the velocity distribution profile of a population of MC-1 when exposed to a higher temperature enables the targeting of hypoxic zones distributed throughout the tumor volume as observed in Ref. [4]. This is a requirement if self-replication of the MTB in the tumor volume is not desirable due to several factors including but not limited to the uncontrolled increase of the amount of toxins. Unless self-replication is possible, which is unlikely for artificial microrobots, the velocity profile must also decrease accordingly. This can be done using a propulsive system that is affected by the human internal temperature or by adding the required circuitry in the FSM depicted in Figure 1E.

## 3. Oxygen Gradient Detector (OGD) and Propulsion System Interface (PSI)

**Figure 2:**
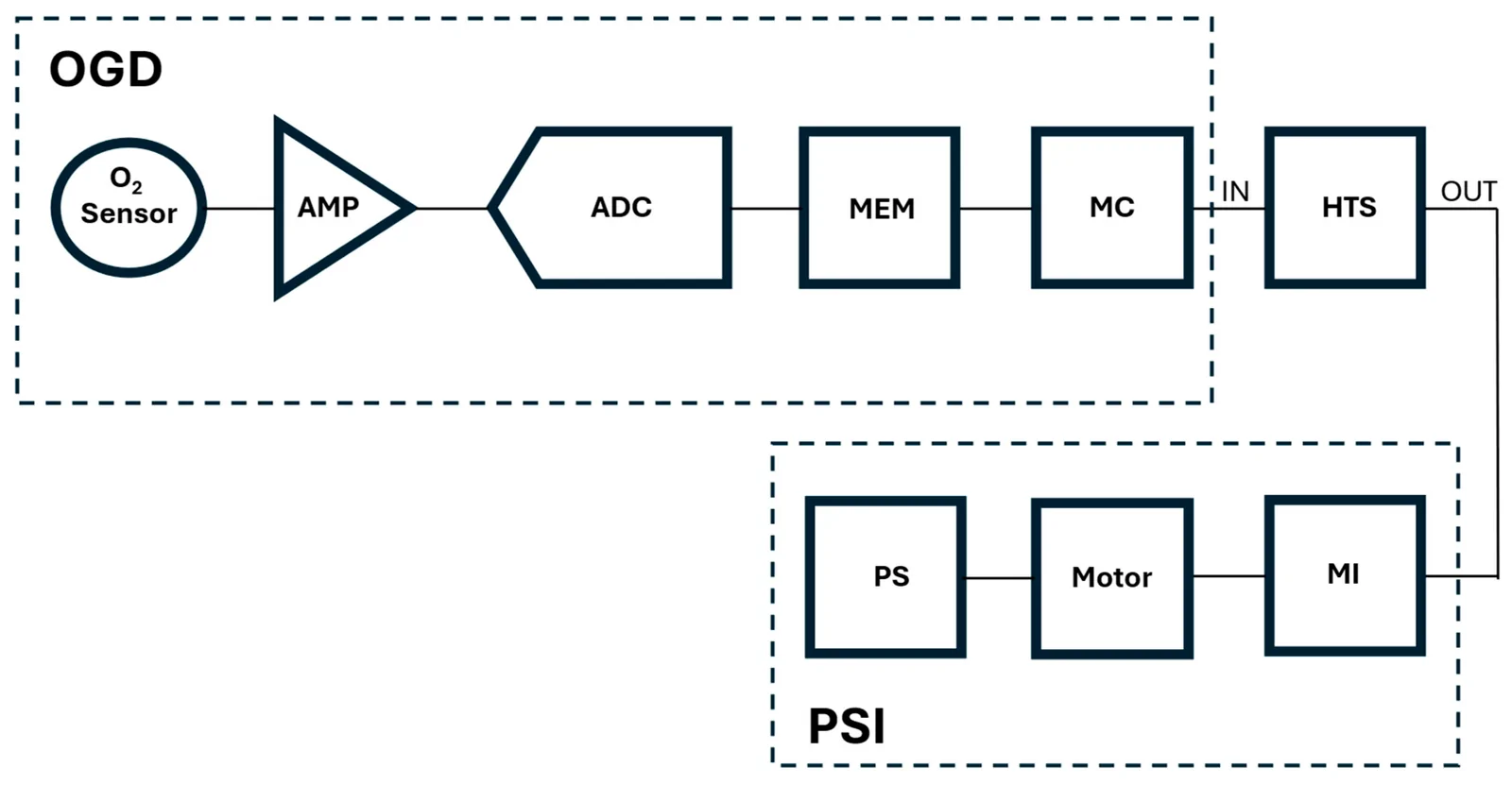
Simplified block diagram of the OGD and PSI.

The oxygen gradient detection system would most likely be composed of an oxygen sensor, an amplifier unit (AMP) acting as a preconditioning interface preceding an analog-to-digital converter (ADC) followed by a memory unit (MEM) and a magnitude comparator (MC) for IN, as depicted in the OGD block in Figure 2. Due to the length restriction of the present paper, only a general description of the following electronic components is provided.

At the micron scale, O_2_ sensors generally rely on one of two transduction mechanisms: optical or electrochemical. The requirement for fiber-optic tips and lasers preclude the use of an optical-based O_2_ sensor. Another option is an electromechanical microsensor, also being referred to as an amperometric ultramicroelectrode (UME) [10]. For UME, a potential is applied to a working electrode typically made of platinum or gold, generating an electrical current which is directly proportional to the partial pressure of oxygen. For instance, such a UME could be implemented using ultrathin platinum wires sealed in glass capillaries with exposed active diameters less than 1 micron, hence providing exceptional temporal resolution with possible response times in the milliseconds due to the microscale diffusion layer. An engineering challenge among others would be related to the signal-to-noise ratio (SNR). As the sensor surface area shrinks to the square of the micron radius, the absolute signal of picoamperes of current generated being so small, it would require an excessively low noise amplifier (AMP).

To simplify the circuit, reduce power consumption, and achieve a higher level of miniaturization, the number of bits of the ADC should be kept to a minimum without lowering the resolution below a threshold that would prevent efficient targeting of tumor hypoxic zones. A MEM unit used to memorize the preceding digital oxygen sample for one CLK cycle, follows the ADC. Such MEM unit can be made of simple DFFs, with the number of DFFs corresponding to the number of bits selected for the ADC. The HTS input IN is then determined using a classical magnitude comparator (MC) used to differentiate the output of MEM with the new oxygen sample prior to replacing the value of MEM with the last sample.

The resulting output (OUT) of the HTS is fed to what is being referred to here as the propulsion system interface (PSI) consisting of an optional motor interface (MI), a motor, and a propulsion system (PS). MI can be a very simple circuit that is able to translate the OUT signal to command signals to the motor if required.

Semiconductor technology is still evolving towards denser circuitries, allowing extreme miniaturization for the benefit of microscale ‘intelligent’ robots, and suggesting that the circuitry related to the FSM, OGD, and PSI could potentially be implemented within dimensions in the range of a few micrometers if relatively large components such as capacitors can be managed accordingly. Static and dynamic power requirements would depend on many design factors. But the fact that the system operates at frequencies in the order of a few Hertzs translates to a significant reduction in dynamic consumption. Furthermore, with operational time requirement of a few minutes to less than 30 min at most depending on travel velocity, distance to cover, and physiological environmental characteristics, further minimization of the overall size of the power source is allowed. Nonetheless, one can expect consumption in the order of tens to a few hundred mA at best with an adequate voltage, meaning that the overall size for an integrated power source is expected to be relatively significant. Looking at the rate of progress in battery technology, it is not expected to be viable without a significant technological breakthrough. To put that in perspective, the smallest form of energy storage, such as a hybrid electrochemical energy storage device based on parallel array of nanowires, showed reversible areal capacity of ~3 µAh/cm^2^ at a current rate of 0.03 mA/cm^2^ [11]. Options for wireless power are extremely limited and unlikely at such a scale and when operating in deep physiological regions. Besides all these issues, only the interconnections to the electronics and other components and the assembly alone would most likely increase the overall dimensions of the robot.

The motor in MTB consists of a rotor within a stator providing propelling thrust exceeding 4 pN prior to injection in physiological spaces, a design much like engineered larger motors. But implementing it at such a small scale while maintaining a sufficient level of performance represents a significant challenge. Indeed, there are claims about the recent smallest micromotor showing dimensions of 400 µm in outer diameter and 800 µm in length [12], exceeding by far the overall dimensions of the microscale robot.

Chemical reactions providing reversable jet-like thrust controlled by the OUT signal of the HTS could potentially be a simpler alternative, although still very challenging at such a scale. But in all cases, the PS must be designed to be efficient in low-Reynolds conditions, like the flagella used by the MC-1.

One potential option to avoid the implementation complexity of the OGD and PSI is the use of heterogeneous particles [13] such as synthetic Janus particles. A classic design involves a polymer microbead coated on one hemisphere with a catalyst such as platinum, manganese dioxide, or enzymes like glucose oxidase. When placed in a chemical medium, the catalytic side reacts with a fuel source (like a trace of hydrogen peroxide or glucose gradient), generating an asymmetrical local chemical or bubble gradient. This asymmetry creates a local fluid flow around the particle via diffusiophoresis, propelling the particle forward. By tuning the surface chemistry, these particles can be designed to alter their speed or tumbling frequency based on local oxygen levels. When they hit a hypoxic zone, the change in local reaction kinetics traps them, causing them to accumulate where oxygen is depleted. However, the MTB’s superior microspatial penetration, and ability to sample the environment and make corrective steering accordingly, in addition to the navigation capability in deep and complex 3D micro-physiological structures, imposes some real challenges for the Janus approach to compete with. At present, the strong dependence of Janus particles on high fuel concentrations and the variability of substrate availability in tissues often lead to insufficient propulsion for efficient transit in tumoral tissue, hindering clinical translation. Nonetheless, potential advances in synthetic self-propulsion and gradient-based motion could allow Janus particles to become an alternative worth consideration in the future.

## 4. Therapeutic Payloads and Release Mechanisms

Since hypoxic zones are generally located from approximately 70 to 180 µm from the tumor vasculature, it is required that the payload concentration delivered in hypoxic areas be sufficient to allow the required drug concentration to treat the further located cancer normoxic cells adequately. Although the high potency of a drug molecule, such as SN-38, enables a reduction in the payload required, the small surface area of such a microscale robot limiting the total payload that can be carried per microrobot must be compensated by a sufficiently large population of drug-carrying microrobots. The exact number depends on many factors, including but not limited to the cancer indication, transcription factor, the drug used, etc. To provide an idea of the number of microrobots required, a study [14] found that at 200 ng mL^−1^ of SN-38, wild-type cells began to undergo apoptosis but not to the full extent. To put these data in perspective, a dose of 200 ng mL^−1^ of SN-38 is equivalent to 10 × 10^6^ MTB-based microrobots corresponding to 10 µL of injectable per mL of tumor tissue with a therapeutic dose concentration of 20 µg mL^−1^ of SN-38 and a concentration of microrobots of 1 × 10^9^ microrobots per mL, meaning that a minimum of 10 × 10^6^ microrobots or 10 µL of microrobots would be required per cm^3^ of tumor tissue to achieve a noticeable level of apoptosis that is still below the maximum apoptosis level. Notice that this number does not account for the added immunotherapeutic effects.

Each microscale robot must be able to not only carry sufficient therapeutic payload to reach further located cancer normoxic cells with the required dose through a sufficiently high diffusion gradient obtained with the cumulative payload of a concentrated population of microscale robots, but also release the therapeutic payload when reaching the target sites, such target sites being here the tumor hypoxic zones. Such release allows more efficient diffusion through a lower molecular mass compared to the much higher molecular mass when the therapeutic payload is still linked to the bacterial cell or an artificial version, the latter preventing the therapeutics from reaching further located cancer cells from the hypoxic zones especially with the negligeable convective flows caused by high interstitial pressure in such tumoral environments.

Beside physical approaches providing relatively weak links such as electrostatic interactions, to name but one example, the main alternatives to strongly attach such payloads to bacterial cells or artificial microscopic robots include but are not limited to the use of properly coated lipid-based encapsulations such as liposomes and micelles, biodegradable polymers such as PLGA or PLA, or the use of a linker. Gram-negative bacteria such as the MC-1 are known to have liposaccharide (LPS) at the surface of the cells. Such a protective shield for the bacterial cells provides a dense surface with molecular characteristics that can be used for attachment. The endotoxin LPS is also a pathogen-associated molecular pattern (PAMP) [15] that triggers an immune response [16], supplementing the effect of the therapeutic payload to yield an immunotherapeutic impact. But although LPS allows an innate immune supplemental therapeutic impact, LPS can also trigger a septic shock. An attenuate version of the gram-negative MTB could be envisioned but within certain limits since it could impact LPS-based attachments. This suggests that targeting and retention in hypoxic zones following intratumoral injections with the minimum quantity of gram-negative MTB would be the most appropriate and safest approach for clinical applications. Because the quantity of therapeutics carried by each MTB is constrained by the small surface available on the cell being characterized with a diameter between 1 and 2 µm, allowing efficient intercellular displacements, there are calls for compensating such surface limitations with a payload based on a drug molecule with much higher potency; hence, this allows the reduction of the number of MTB or microscale artificial robots required and therefore, the risk of sceptic shocks while facilitating intratumoral injections due to the need of a smaller injected volume.

SN-38 [17] is only one example among other potential molecules. SN-38 (7-Ethyl-10-hydroxycamptothecin) is a topoisomerase I (TOP1) cytotoxic drug and an active metabolite of the prodrug irinotecan (CPT-11). Compared to CPT-11, SN-38 has been reported to exhibit up to 1000-fold more cytotoxic activity against various cancer cells, hence preventing it from being delivered systematically in its actual form in sufficient amounts for cancer treatments. Released SN-38 from MTB or an artificial microrobot, being membrane-permeable, also yields a bystander effect where SN-38 can diffuse out of the targeted cancer cell and kill neighboring cells, including those that may have low or no expression, increasing the treatment efficacy for heterogeneous tumors.

Liposomal formulation of SN-38 covalently conjugated to the surface of MC-1 has already been proven to be feasible [18], although the efficient encapsulation of SN-38 in liposomes remains a challenging process. Embedding SN-38 in biodegradable polymeric particles being attached to the surface is also possible but the limited loading capacity might be an issue, depending on the potency of the drug molecules being used. There are different conjugation techniques available. Prior liposomal attachment to MC-1 relied on carbodiimide chemistry exploiting the functional inherent moieties (amine group) in the bacteria membrane without chemical manipulation of the bacteria, hence maintaining a suitable level of bacterial motility. The merits of this MTB-liposome bioconjugation include direct attachment, high strength bond, high coupling efficiencies, and reproducibility. Furthermore, it can be used for a wide range of particles including polymeric particles. Covalent or affinity conjugation techniques such as biotin-avidin or maleimide-thiol require chemical manipulation of both bacteria and nanoparticles, exposing MTB to chemical stresses that may impact their functionality and viability. But unlike live microorganisms, such chemical stresses would most likely be acceptable for artificial or synthetic microrobots. The release mechanism relies mostly on enzymatic degradation yielding a specific release profile. But another viable and popular option that avoids encapsulation of the payload is the use of a linker.

With a linker, generally, to successfully attach SN-38 to LPS, a trifunctional crosslinker architecture is needed. Such a molecular bridge requires an LPS-reactive handle on one hand, a bio-reductive, hypoxia-sensitive core in the middle, and a spacer tied to the 10-hydroxy (10-OH) group of the SN-38 molecule on the other hand.

Core oligosaccharide and Lipid A modifications in many Gram-negative bacteria contain primary amines like phosphoethanolamine, allowing a non-drug end of the linker with an NHS ester or a Sulfo-NHS ester to form stable amide bonds with the LPS surface. An oxidative approach could also be envisioned. Indeed, mild periodate oxidation of the cis-diols on the outer O-antigen sugars of the LPS generates active aldehydes, allowing the use of a hydrazide or alkoxyamine terminated linker to achieve covalent oxime/hydrazone coupling.

On the other end of the linker, we generally cannot attach a hypoxia-cleavage trigger directly to SN-38 without ruining its therapeutic index since when the linker breaks, any leftover chemical tail attached to the drug molecule would most likely render it inactive. A potential solution would be to embed a PABC (p-aminobenzyloxycarbonyl) [19] or a modified benzyl carbamate spacer between the hypoxia trigger and the 10-OH position of SN-38.

The tumor hypoxic core is rich in overexpressed reductive enzymes (reductases) because the lack of molecular oxygen prevents normal competitive oxidative pathways. Two main chemical groups are highly stable in oxygenated regions but cleave rapidly under hypoxia, Azo-Benzene linkers [20] and Nitroimidazole derivatives. With the former, when the conjugate reaches the hypoxic zone, upregulated azoreductases selectively reduce the azo group into electron-rich aromatic amines, breaking the central double bond. Single molecule nano-prodrugs of SN-38 favor azobenzene because it provides a clean, predictable cleavage cascade. Once the azo bond is split, it triggers a spontaneous self-elimination that releases completely unmodified active SN-38.

As for Nitroimidazole derivatives [21], under normoxic conditions, 2-nitroimidazole is continuously oxidized back to its native state. Under hypoxia, oxygen cannot compete, allowing intracellular nitroreductases (NTR) to single-electron-reduce the nitro group (-NO_2_) into a highly unstable aminoimidazole (-NH_2_) resulting in a cleavage cascade where the electron-donating power of the new formed amine initiates a 1,6-elimination or a cyclization–elimination sequence, fracturing the adjacent carbamate or ester bond to liberate the drug payload.

Using the same strategy as selecting an adequate FDA-approved drug (e.g., SN-38) for the payload instead of developing a new drug molecule with the risks involved during the development phase, another approach that could be considered more predictable in terms of development is to rely on the use of an already commercially available FDA-approved linker for SN-38. A recent review by WuXi AppTec, dated 30 January 2026, identifies 21 approved linkers (the list of those linkers is depicted in Table 1 of this recent review by WuXi AppTec) [22]. From the same list, only the linker CL2A [22] commercialized under the trade name Trodelvy has SN-38 as payload, hence significantly reducing the development efforts needed and associated potential risks, leaving only the need to put most efforts towards LPS attachment, which can be a relatively easy task compared to the implementation of other specific functional blocks in an artificial version of the bacterial robot.

The CL2A linker’s primary cleavage mechanism is pH-dependent hydrolysis of its benzyl carbonate bond which is accelerated in acidic environments such as the tumor microenvironment (pH ~6.0–7.0), and cellular compartments like endosomes (pH ~5.5–6.0) and lysosomes (pH ~4.5–5.0), while premature cleavage has been observed at pH levels as high as ~7.4 (such as in blood systemic circulation) [23], resulting in the release of the active drug SN-38. According to previous study showing a half-life of greater than 36 h at pH 7.4, a representative half-life of the accelerated hydrolysis in the tumor microenvironment would be less than 36 h but still slower than acid-catalyzed hydrolysis inside the cancer cells. Therefore, although the release of SN-38 from the MC-1 bacteria or an artificial microscale robot would be possible within an acceptable delay, subsequent injections may yield slower hydrolysis following the treatment of hypoxic cells.

These approaches and techniques can be relatively easily reproduced for artificial and synthetic microscale robots. Furthermore, the potential chemical stress affecting the motility of a microorganism-based robot is typically not an issue for artificial and synthetic implementations, adding further flexibility in selecting an adequate approach.

## 5. Artificial Representation of the Magneto-Aerotactic Bacterial Robot

**Figure 3:**
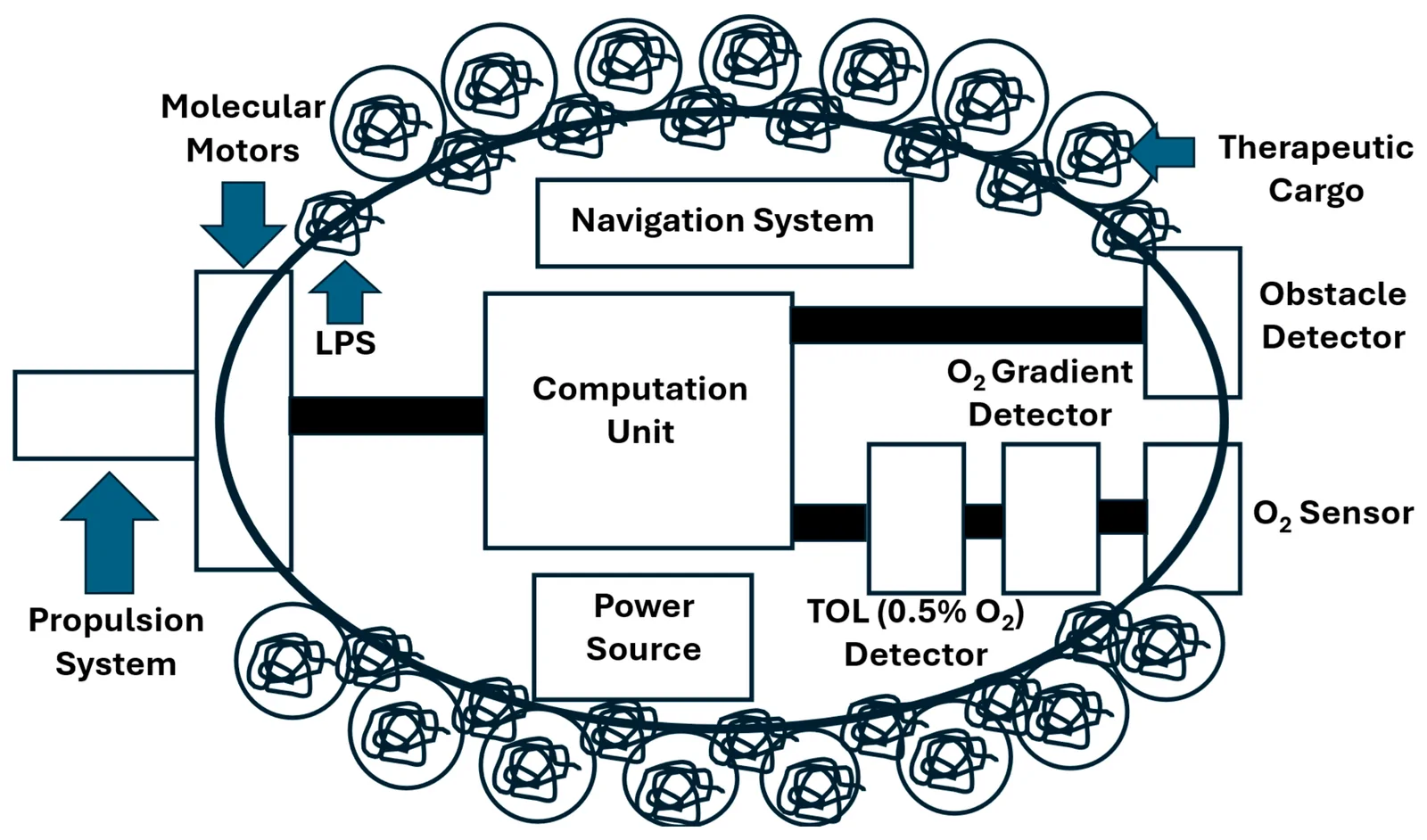
Simplified block diagram of the polar magneto-aerotactic bacterial robot.

A simplified schematic representing a polar magneto-aerotactic bacterial robot as a potential artificial implementation is depicted in Figure 3. The navigation system shown in the diagram is used to guide the microscale robot along the direction of the lines of magnetic field. Such a navigation system can be a simple anisotropic magnetic structure acting as a magnetic compass needle, similarly to the chain of magnetosomes embedded in MTB. Such a component is therefore considered highly feasible for the implementation of an artificial or synthetic version of the bacterial microrobot.

The computation unit acting as the brain of the microrobot is the two-states FSM referred to as the HTS, with an example of the corresponding circuit shown in Figure 1E. The simplicity of such a circuit suggests its potential integration at such a small scale. The decisions made by such a computation unit or HTS depend on the level of oxygen gathered through an O_2_ sensor and an O_2_ gradient detector. As described in Section 3, the implementation of these components in an artificial robot can be extremely challenging and unlikely in the near future, especially if one is considering the issue related to the power supply, which is also a constraint with regard to the propulsion system. The oxygen sensor with the gradient detector and the TOL detector, being part of the OGD as depicted in Figure 2, pose a real challenge when implemented with conventional electronics, especially taking into consideration the power requirement. The optional obstacle detector would enhance the capability of the robot to penetrate deeper in the complex tumor microenvironment (TME). The description of all these electronic components suggests that in a shorter term, adopting a synthetic implementation such as the use of Janus particles may be a more suitable approach to compete with such types of biological microrobots, although much work is needed to achieve the level of performance of such MTB-based robots.

As mentioned earlier, the implementation of an efficient miniature motor mimicking the molecular motor of MTB is a real challenge. While power only needs to be available for a relatively short time, seconds or minutes at most, providing sufficient and reliable power at such a small scale and type of artificial implementation is indeed a major obstacle. As mentioned earlier, this alone suggests that propulsion, like the one used for Janus particles for instance, being also efficient at low Reynolds, could be a more promising approach to compete with a biological version.

Also as mentioned earlier, the encapsulation and attachment of the therapeutic payload being in the order of 0.0002 ng of SN-38 per MTB-based microrobot, as well as the release mechanisms possible for MTB-based robots, can also be implemented for artificial and synthetic microrobots. For a synthetic microrobot based on a Janus particle, for example, the therapeutic payload could be embedded in a propelled biodegradable or degradable implementation of the particle.

Clinical translation also relies on the safe use of such drug-loaded microrobots. In the systemic response to MC-1 cells, although no human trials have been conducted yet, the fact that no significant differences have been observed in the expression of inflammatory cytokines in the blood for PBS and MC-1 injections both in A/J and in C57BL6 mice [4] could be interpreted positively for interventions in humans. Although the present trend in artificial medical microrobotic is principally aimed at avoiding the immune system, another strategy used by MTB-based microrobots is to trigger the immune system to achieve a complementary immunotherapeutic response from lipopolysaccharide (LPS) and flagellin which could also be possible for artificial and synthetic microrobots.

Despite the many advantages of the biohybrid microrobot depicted in Figure 3, such implementation also comes with some issues and constraints that must be evaluated against the benefits of more efficient treatments through the integration of such specific embedded functionalities not readily possible with other approaches. For instance, the use of microorganisms, such as the MTB-based microrobot shown in Figure 3, imposes restrictions in the synthesis process to avoid stresses that could impact the viability of the microrobots prior to a medical intervention. Artificial and synthetic microrobots are generally more tolerant to such potential stresses. In the case of MTB-based microrobots, the swimming velocity decreases gradually when exposed to human body temperature prior to being eliminated through phagocytosis. Although the velocity profile is generally adequate to treat tumors, it restricts the injections to be performed closer to the tumor or intratumorally. Self-propelled synthetic microrobots such as Janus particles share a similar restriction which is typically not the case for microrobots relying on an external source for propulsion. Although using the latter in a physiological microenvironment may prove extremely challenging, it remains a superior alternative for operations in larger physiological spaces, especially the arterial network, allowing the inducing of larger propulsive forces that are far beyond the propelling forces of MTB-based microrobots, making it possible for them to operate in environments characterized with a higher blood flow velocity.

## 6. Conclusions

The advantages of microrobots for treating cancer becomes more evident when specific capabilities can be implemented at such a small scale. As such, the use of microorganisms such as magneto-aerotactic bacteria enables the implementation of microscale robots with such embedded capabilities that are hard to achieve using artificial or synthetic components alone. Although the use of conventional electronics to mimic the capabilities of magneto-aerotactic bacterial robots, due to many factors including but not limited to power issues, at such a small scale, is unlikely, at least in the near future, this suggests that synthetic approaches such as the use of Janus particles may be more promising in the shorter term to compete against biohybrid microrobots. Nonetheless, the use of magneto-aerotactic bacteria remains a viable and powerful approach for cancer therapy with many advantages such as self-reproducibility, to name but only one significant advantage. Although synthetic approaches can also benefit from the same attachment and release mechanisms that could be used for biological microrobots, substantial efforts still necessary before the capabilities of such synthetic versions become equivalent or surpass the capabilities of magneto-aerotactic bacterial robots for tumor treatments. It is also true that synthetic methods will evolve, but the same will likely happen for biohybrid robots as well, especially with advances in synthetic biology. Looking forward, it becomes clear that biology and especially the use of synthetically improved living microorganisms will play a great role in the development of future microscale robots dedicated to cancer treatments, enabling complex tasks and functionalities that will be challenging for artificial and synthetic versions to compete with. Nonetheless, artificial and synthetic implementations will remain superior options for specific tasks and physiological environments such as operations in larger blood vessels where the flow is too high to enable effective operations by microorganism-based robots.

## Data Availability

No new data were created or analyzed in this study. Data sharing is not applicable to this article.
